# Communication Technologies for Interoperable Smart Microgrids in Urban Energy Community: A Broad Review of the State of the Art, Challenges, and Research Perspectives

**DOI:** 10.3390/s22155881

**Published:** 2022-08-06

**Authors:** Gogulamudi Pradeep Reddy, Yellapragada Venkata Pavan Kumar, Maddikera Kalyan Chakravarthi

**Affiliations:** School of Electronics Engineering, VIT-AP University, Amaravati 522237, Andhra Pradesh, India

**Keywords:** communication standards, communication technologies, interoperability, interoperable smart microgrids (ISMs), LoRa (Long Range), LPWAN (Low-Power Wide Area Network)

## Abstract

In modern urban energy communities, diverse natured loads (homes, schools, hospitals, malls, etc.) are situated in the same locality and have self-electricity generation/management facilities. The power systems of these individual buildings are called smart microgrids. Usually, their self-electricity generation is based on renewable energy sources, which are uncertain due to their environmental dependency. So, the consistency of self-energy generation throughout the day is not guaranteed; thus, the dependency on the central utility grid is continued. To solve this, researchers have recently started working on interoperable smart microgrids (ISMs) for urban communities. Here, a central monitoring and control station captures the energy generation/demand information of each microgrid and analyzes the availability/requirement, thereby executing the energy transactions among these ISMs. Such local energy exchanges among the ISMs reduce the issues with uncertain renewable energy and the dependency on the utility grid. To establish such useful ISMs, a well-established communication mechanism has to be adopted. In this view, this paper first reviews various state-of-the-art developments related to smart grids and then provides extensive insights into communication standards and technologies, issues/challenges, and future research perspectives for ISM implementation. Finally, a discussion is presented on advanced wireless technology, called LoRa (Long Range), and a modern architecture using the LoRa technology to establish a communication network for ISMs is proposed.

## 1. Introduction

**Figure 1:**
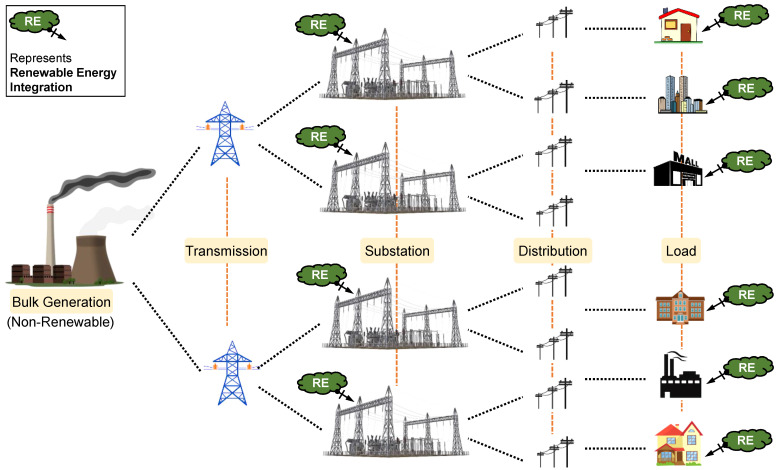
Renewable energy integration in the present power system scenario.

Present-day challenges in the energy sector, such as rising electricity generation prices, losses in energy transmission, fossil fuel depletion, environmental concerns, poor efficiency, higher installation cost/time, etc., have motivated the search for alternative and local power generation systems. Further, the unrelenting growth of urbanization in the energy sector has greatly increased the utility grid burden, which causes frequent grid outages. All of these factors have laid a path for the deployment of distributed energy systems to reduce the dependency on the utility grid. So, the traditional central utility grid is supplemented with renewable energy-based local generating plants [1]. Renewable energy can be integrated at the substation level (usually large in terms of capacity) or building level (usually with a small capacity) in the power system, as shown in Figure 1 [2]. These kinds of micro-level power generation capacities using the renewable energy sources that are connected to buildings at the distribution voltage level are called “microgrids” [3]. Further, a steady change in the expectations and comforts of electricity consumers day-by-day demands the system to be more responsive.

**Figure 2:**
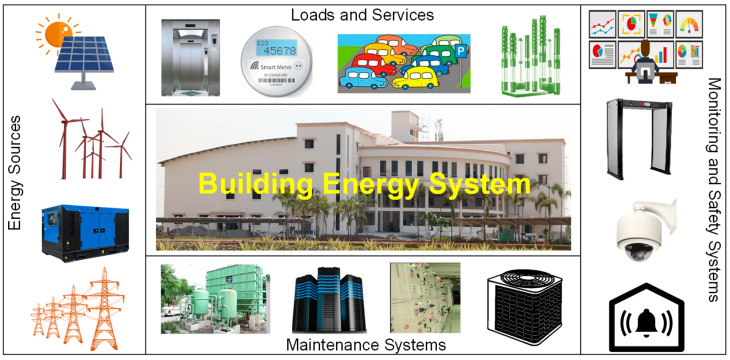
Constituents of a smart microgrid associated with a building.

Thus, these microgrids are presently evolving as intelligent local grids, called “smart microgrids”. These provide unique features to the electricity consumers, such as demand-side management by peak load curtailment or shaving, deregulated marketing (dynamic pricing options with real-time trading), forecasting for contingency readiness, demand response, outage management, energy conservation, energy efficiency enhancement, etc. [4]. To maximize the efficiency and productivity of a smart microgrid, the integration of various components in and around a building plays an important role [5]. These include various types of energy sources, loads and service equipment, monitoring and safety systems, and maintenance systems, as shown in Figure 2, where information can be exchanged through wired or wireless communication. However, the intermittent behavior of renewable energy sources lessens the fruitfulness of the microgrids by complicating their responses to real-time loading.

### 1.1. Need and Opportunity for the ISMs

Microgrids are ecologically clean and green, deregulated, and decentralized, and can reduce the burden on the utility grid if they are operated reliably. However, these systems possess unsteady generation capacities due to the dependency on uncertain environmental factors. Due to the unstable nature of the microgrids, central power grid outages have continued. In such scenarios, the integrated/combined operation of multiple smart microgrids in a locality (named ISMs—interoperable smart microgrids) allows well-thought-out contact between suppliers and customers, which enables their operating approaches to be both more versatile and sophisticated.

On the other hand, during the early days of urbanization, urban communities were formed by a group of homogeneous buildings (buildings with similar load profiles), so they cannot share energy sources among themselves. However, there is a paradigm shift in the modern urbanization scenario: communities are forming through heterogeneous buildings (buildings with diverse load profiles), such as those of industries, apartments, hospitals, universities, markets, malls, theatres, etc. As all these buildings may not have the same load at any one time, these new formations make it possible to share resources. This is a new opportunity that needs to be adapted by researchers to interoperate various microgrids to enhance their fruitfulness. However, the major challenge lies in finding an optimal unit commitment strategy to economically dispatch the generated energy various microgrids [6].

Additionally, the modern smart grid initiatives suggest including various features such as central monitoring and control unit (CMCU) operations, demand response, effective generation-load balancing (energy management), ICT (information and communication technology) for continuous data monitoring and effective data processing, forecasting for contingency readiness, emergency alerting, etc.

Hence, to address the abovementioned issues and to match the requirements of modern smart grid initiatives, this paper proposes the implementation of ISMs in an urban energy community. This interoperable or integral operation in a locality can enhance energy availability and generation capability. This provides a new opportunity to effectively address the issues of microgrid deployment as the generated energy can be collectively utilized rather than utilized alone. Further, this local management of available energy can reduce the dependency on the utility grid energy.

### 1.2. Problem Statement and Article Structure

There are some review papers published in the literature that discuss various aspects of smart grids’ development. Table 1 summarizes all such works.

**Figure 3:**
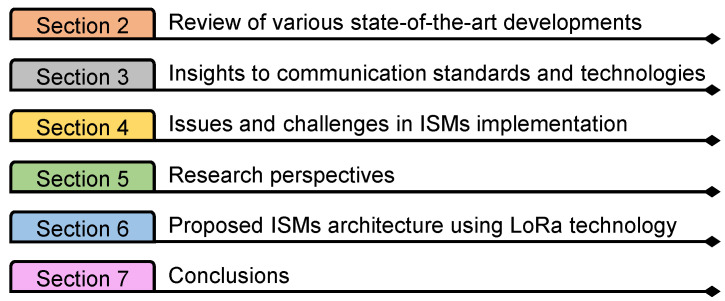
Organization of the paper.

From these works, it is understood that most of the research work focuses on smart grids; few have addressed microgrids. Additionally, few works have discussed the standalone communication technologies, which can help us understand the communication concepts. So, it is evident that ISM-based analysis has not been conducted so far. For this purpose, this paper presents a comprehensive investigation into the state-of-the-art developments, insights into communication standards, technologies, and issues/challenges present in ISM deployment. This paper also discusses key future research perspectives followed by a proposed ISM architecture using LoRa (Long Range) technology. All of these are covered in various sections of the paper, as shown in Figure 3.

## 2. Comprehensive Review of Various State-of-the-Art Developments

This section discusses the worldwide footprint of smart distribution grids and initiatives in the Indian power sector, along with a review of research works related to both the macrogrid and microgrid levels.

### 2.1. Worldwide Footprint of Smart Distribution Grids

Smart distribution grids are evolving as a potential power system composition in many countries, created by integrating renewable energy resources into the utility grid. Some of the recent projects are: CFCL BlueGENs UK grid, Ecogrid Project, ERIGrid, Smart Power Hamburg Project, Danish Edison Project, Samso, Ei Hierro, GRID4U, and Power Matching City Projects (in Europe); Toyota city project, Kitakyushu city project, and Hanian (in Asia); PRICE, STAmi, ISOLVES: PSSA-M, ELECTRA, Smart Grid, Hyllie, Model City, Manheim, Vendee, Nice Grid, e-GOTHAM, Arrowhead, and NINES [10,14]. Apart from these, many other countries have also started or deployed smart grid projects. Table 2 gives a summary of all such worldwide initiatives.

### 2.2. Initiatives in the Indian Power Sector

Apart from the projects mentioned above, there are several other projects related to the smart grid being implemented across India as given in Table 3. Importantly, the Govt. of India approved 14 Smart Grid pilot projects in India. The Ministry of New and Renewable Energy (MNRE) is working towards having a solar installed capacity of 100GW by 2030 and 200GW by 2050 [2].

### 2.3. Review of Research Works—Macrogrid Level

This section presents various key works in the literature related to macrogrid communications, which are summarized in Table 4. IEEE 802.15.4g is an important standard for Smart Utility Communications Networks (SUN). This standard helps to establish a common set of rules over the globe for the interoperability of smart grids. This is a PHY layer and three modulation techniques MR (multi-rate and multi-regional)-FSK, MR-OQPSK and MR-OFDM are discussed. Frame formats help us understand IEEE 802.15.4e amendments, which is a MAC layer. There are two types of information elements (IEs) defined in 802.15.4e, header information elements and payload information elements. To have a complete Advanced Metering Infrastructure (AMI), completing the design of PHY and MAC layers is essential. However, this is limited to the outline of standard descriptions such as PHY 4g and MAC 4e [42].

State-of-the-art communications infrastructure, concerns and applications in the smart grid are described in [43]. A comparison table between the existing grid and the smart grid is made, demonstrating the importance of the smart grid. Nearly 75 standards are available for the building infrastructure of smart grids. The ability of WSNs (wireless sensor networks) to facilitate the realization of the smart grid is also discussed [43]. Three major challenges are addressed: guaranteeing uniform interoperability, cognitive accessibility to unlicensed radio spectra and cybersecurity. In summary, WSNs, wireless communication technologies, challenges in interoperability, unlicensed radio spectra, and cybersecurity are discussed.

The SGIRM (smart grid interoperability reference model) is a conceptual smart grid architecture model [44] and is described in three aspects: (1) electrical; (2) communications; and (3) IT. The level of assurance in communication links is also classified as Tier 1 (critical), Tier 2 (important), and Tier 3 (informative). It highlights the idea of a Unified Key Management Function (UKMF). Security and key management issues are discussed. However, there are many standards on interoperability to be covered. Energy management plays a significant role in the electric distribution network [45]. The data stream of each intelligent energy management (IEM) can be in any of the three modes, i.e., the direct transmission mode, traditional relaying mode and D2D-assisted relaying mode. An architecture using D2D communication along with the relaying techniques is presented in [45]. This minimizes the overall rate of information loss and peak bandwidth demand for latency-sensitive smart grid communications. However, some assumptions were made around relay nodes and base stations, which may affect the efficacy of the system.

One of the smart grid’s major advantages is bidirectional communication [46]. Communication between different deployed smart meters and a gateway is very crucial. Malicious users could potentially access the data. Thus, a lightweight authenticated communication (LAC) scheme ensures safe data transmission between smart meters and gateways. The LAC has lower storage and communication costs and achieves confidentiality, honesty, authentication in real time, and attack–resistance replay.

Different communication technologies (wired and wireless) (PLC, DSL, optical communication, ZigBee, Wi-Fi, Wi-Max, GSM, Sigfox, Narrowband-Internet of things (NB-IoT), LoRa, etc.) are discussed [14]. These can be used in the context of smart grids with an emphasis on smart metering LV applications. Various smart meter projects around Europe are also discussed. Nevertheless, initiatives based on the MV smart grid have not been discussed.

The potential of WSNs (wireless sensor networks) is utilized for smart girds [47]. The presented Vehicular Ad hoc Network (VANET) is used to gather smart meter data. The data flow is from a house with a wireless automatic meter reading (WAMR) to a bus stop and these data are transmitted to the bus. However, the smart meter should have IEEE 802.11p compatibility and houses should be within 1000 m of the bus stop.

In communications, the spectrum plays a significant role. The utilization and reuse of the available spectrum are very crucial. A new approach for the sharing of the spectrum in smart grids is addressed in [48]. Applications are categorized into three types: Class 1, Class 2, and Class 3. The throughput is increased by the suggested application class (priority-based communication strategy). To explain this concept, a case study of the IEEE 14-bus power grid is also mentioned. However, the distance was not given importance, which may influence the proposed method.

National power utility company (PLN), Bali, has recently introduced Advanced Metering Infrastructure (AMI) by replacing traditional meters with smart meters [49]. Different LPWAN technologies (Sigfox, NB-IoT, and LoRa) were discussed to collect data from smart meters. The PLN Bali sends a request to the regulator to use Sigfox or NB-IoT as a telecommunications provider. LoRaWAN is used as a solution to collect the data from the houses in the 1000 m radius. PLN could achieve a 100% success rate. However, detailed metrics for the actual data are not described.

The smart sensor can supply real-time data and the grid status for multiple operations [50]. The integration of these smart sensors for smart grids plays an important role in interoperability. The general model of smart sensors for smart grids is discussed. PMU-based and MU-based smart sensors are also described. Various interfacing standards such as IEEE 1815, IEC 61850-9-2 and 61869-9, IEEE C37.118, and IEEE 1451 are discussed. An interoperability test system was developed for PMU-based smart sensors, and testing was carried out. This requires high-accuracy timing and time synchronization.

NB-IoT is one of the latest technologies in the area of LPWAN, and works in a licensed band (country-specific) [51]. With NB-IoT, the desired features, such as being long-range, low power, and high capacity, can be achieved. A high Quality of service (QoS) can be achieved with NB-IoT, whereas it is usually difficult to achieve with other technologies. Qualitative metrics (security, scalability, flexibility, and availability) and quantitative metrics (latency, frequency range, reliability, and data rate) are also discussed. With these metrics, it was concluded that NB-IoT is a useful technology, but it is costlier for real-time deployment as it requires a proprietary license and large infrastructure.

The importance of wireless communication technologies (especially LoRa) in establishing communication between smart grids is discussed in [52]. Path loss, the shadowing effect, and multipath fading are also discussed. Further, a formula was derived for calculating the distance between the transmitter and receiver. Here, the work is two-fold: one is a theoretical approach and the other is a practical approach. The authors used 4 transmission parameters, 2 environmental parameters, and 1 parameter for randomness to calculate the distance. In earlier works, researchers mainly focused on the spreading factor (typically between 7 and 12) as an important parameter which affects the performance of LoRa. However, from the results given in [52], it was concluded that distance, obstacles, and noise are the three most significant factors affecting the performance of LoRa technologies. However, only a few parameters were considered while designing the wireless channel. Further, Saleh Valenzuela channel modelling can be applied and optimum node placement can be focused on.

In smart grid communications, the security threat to data can be from insiders or outsiders [53]. An investigation was previously conducted on various possible security insider threats and the possible solutions were summarized. In addition, a novel hybrid insider threats model was also proposed. However, the construction of rules has to be more detailed.

ICT (Information and communications technology) is very important for smart grids [9]. The major architectural issues, key technologies and infrastructure requirements in smart grids are discussed. The importance of the cloud is also detailed. Various standards such as IEEE, IEC, NIST, ANSI, ITU-T, and SAE are discussed. Future directions using Software-defined networking (SDN), network virtualization, network coding and 5G networks are listed along with the discussion on PHY and application layers.

### 2.4. Review of Research Works—Microgrid Level

The following are the various literature works conducted on microgrid communications. All the key points are summarized in Table 5. The integration of distributed generation units to the conventional grids can tremendously increase the performance of the grids. However, at the same time, this integration may have an impact on the structure of the grid. The use of microgrids is the better solution in this case. A microgrid is a combination of loads, local generations (typically renewable sources), controllers, protection and management systems [18]. Various projects in Europe, Japan, Korea, North America and Australia are detailed. However, there are very few works available on microgrid protection and energy management systems.

Jordon is a developing country with a very small population of around 97 lakhs, and 96% of the nation’s energy originates from oil and gas imports from other countries [54]. Jordon experiences strong global radiation, 2080 kWh/m^2^, with over 300 sunny days in a year. Jordan can make use of solar energy to produce electricity. The concept of a local area grid is discussed, which is to have a single source of power generation (PV) and distribute the energy within the apartments/households that exist in the same building/community. This system consists of four major components: (1) solar panels, (2) inverter, (3) batteries (optional) and (4) intelligent power distribution and control unit (IPDC). This solution provides low-income households with the ability to share the cost of installation while substantially reducing energy bills. Further, various technological advancements in microgrid communications are discussed in [55]. State-of-the-art and future developments are summarized as well. Internet Protocol suite, DNP3, Modbus, IEC 61850 and different wired and wireless physical links are mentioned.

The microgrids operating in a particular environment should operate at the same frequency [56]. If the frequency produced by the grids is different, then there may be a chance of damaging the load. The establishment of frequency synchronization between the grids is discussed by representing each grid as a Linear time-invariant (LTI) system. To accomplish this, a consensus algorithm based on a cooperative control strategy is used. Similarly, the purpose of the research of [57] is to restore the frequency of hybrid lossy microgrids (using a distributed secondary control algorithm). The distributed communication network under consideration has time-varying delays in communication. A stable Lyapunov–Krasovskii analysis methodology is used for the study.

Communication between various distributed generation units in a microgrid is established using Zigbee technology [58]. Each unit has a local controller in addition to the central controller at the microgrid level. The advantage of Zigbee is its low cost and low power consumption, but it works at a very low data rate. To reduce the number of transactions, a data management scheme was proposed. The local (primary) controller, central controller, and network tertiary controller are also discussed. Communication delay is considered one of the important metrics to explain this concept. It is expected for a microgrid to operate in two modes: standalone mode and grid-connected mode [59]. The goal is to incorporate communication and control to facilitate the transition from the standalone to grid-connected mode. The authors presented a security scheme for this purpose and the performance is compared with those of the Rivest–Shamir–Adleman (RSA), Digital signature algorithm (DSA), and Time Valid Hash to Obtain Random Subsets (TV-HORS).

The primary focus of [15] is to review the state-of-the-art research on the microgrids in both islanded mode and grid-connected mode. Reliability, resiliency, and power quality are discussed as the key parameters. Further, various economic issues and other elements of the microgrids are outlined. Further, a review of different architectures (IEEE-1547, ISA-95, NISA, and IEC-61850) was presented in [16]. Some of the key challenges such as the lack of awareness and clarity, technical challenges, etc., are emphasized. Additionally, various retrofitted architectures and topologies to improve the clarity of presentation and perception of architecture IEEE-1547 and redundant architecture to improve the network consistency of the IEC-61850 architecture were discussed.

Distribution system automation using ICT is introduced as a resolution that incorporates all of a distribution system’s essential constituents [60]. The evolution of the automation perspective in needs and technology was discussed. Different sub-systems are listed as process improvement and decision support systems, process optimization systems, communication networks, database management and maintenance systems, and process control and safety systems. The home energy management network (HEMS) plays a major role in the smart grid [61]. There were several problems with the traditional HEMS, such as scalability, reusability, etc. Further, a dynamic home area network (DHAN) is proposed which is an IoT-based HEMS. Unlike traditional gateways, here, a nomadic agent is used to achieve this dynamic nature. Through the experiments, the suggested method could achieve energy savings. 

The installation of a microgrid in Griffith University’s N44 building is discussed in [62]. The communication architecture and data acquisition mechanism were discussed. The communication protocols used were Modbus and TCP/IP. Python was used to access the data and to make them available on the cloud. The use of various hardware components (SunnyBoy Webbox, RedLion data logger, power meters, etc.) was discussed. Similarly, various views of distributed resource system architectures (architectural view, requirements view, conceptual view, concurrency view, and network view) are discussed in [17]. The analysis and refining process were conducted in compliance with the guidelines of IEEE 1547.3TM-2007. Various game theory approaches (cooperative, non-cooperative, one shot, etc.) were used to establish energy-sharing mechanisms within the households in a selected microgrid (in Stockholm) [63]. Utility is a key metric to quantify a household’s payoff from playing a game. The distributed algorithm and Gale–Shapley algorithm are used to establish power-sharing between households. The benefits of this approach are presented in terms of cost savings and emission reductions.

The microgrid consists of three types of power converters, namely, grid-feeding, grid-supporting, and grid-forming power converters. In island mode, these converters should maintain the voltage and frequency in the desired range [64]. A microgrid central controller achieves synchronization in grid-forming power converters. Noise and the synchronization signal delay play a significant role, and can also lead to a quality loss in the grid-forming power converters. The RS-485 communication protocol is used to implement the system. Further, to achieve robustness, the FM signal is used as a synchronization signal. During extreme events (flood, earthquake, hurricane, etc.), the healing time of a microgrid, which is called resilience, is very important. One should have a thorough knowledge of microgrids from end to end to help in this situation. So, the management of networked microgrids for riding through extreme events is essential. In this view, community resilience microgrids are used to share the energy generated by their sources within a community [12,65]. Though it can be used in normal operating conditions, it has great impact in emergency situations. The unique operational characteristics of these microgrids are also presented. Electricity ethnography (a three-month analysis) was conducted at an off-grid village in rural India (1 February–30 April 2016)) [66]. This report discussed how social relations and diverse cultural values influence the exchange of energy between village households. Two types of energy exchanges (energy-sharing and energy-trading) are discussed. The integration of four microgrids with the help of the Internet, i.e., cloud-based service (SaaS), is discussed in [67]. The individual components of the microgrid are connected (IEEE 802.11s-based mesh network) and all these are connected to a gateway. All four gateways are connected to the cloud platform with the implementation of IEC61850 GOOSE messages. Further, a bilevel distributed optimization algorithm was developed using LabVIEW and NS-3.

The design and modelling of intelligent electronic devices (IEDs) based on IEC61850 are discussed for various forms of distributed energy resources [68]. The modelling of IEDs for PV plants, battery systems, diesel plants, wind turbines and controllable loads is discussed. Additionally, the communication services (GOOSE, SV, etc.) are also presented through a real-time system-in-the-loop simulation. Additionally, a review on ICT for microgrids is given in [11]. An evaluation of microgrids, from natural microgrids to dynamic microgrids, was conducted and further suggested using a peer-to-peer communication approach for next-generation microgrids. Similarly, ref. [69] focuses on developing the communication models of solar home systems and smart meters based on the IEC 61850 standards. Simulations are run with a riverbed modeller to evaluate performance. The packet loss of different messages, ETE delay, etc., are discussed. Similarly, a comparison of a P2P (peer-to-peer) energy-sharing mechanism with P2G (peer-to-grid) trading was discussed in [70]. In P2G trading, when the electricity production is higher than the load, the excess PV energy is first used to charge the battery, while in P2P, the excess energy is first used to supply the neighbors in need. The P2P-sharing community contains three types of players: ESC (energy-sharing coordinator), prosumer and consumer. The suggested system is implemented in three stages: (i) two-stage aggregated control (CNLP optimization and control based on rules); (ii) P2P trading; and (iii) assessment. By using assessment metrics such as self-consumption, self-sufficiency, and energy cost, it was concluded that P2P leads to better results than P2G.

A microgrid can be one of three types, DC, AC, or hybrid (AC-DC), depending on the main bus voltage linking [71]. Various schemes, viz., centralized communication-based control, distributed communication-based control, and voltage droop control, is discussed. The use of ICT and allowable latency for different wireless technologies is mentioned. Further, various components of microgrid communication, namely, wired (optical fiber communication, telephone network communication, twisted pair/coaxial cable, and power-line communication) and wireless (GPRS, LAN, Wi-Max, and Zigbee) technologies are discussed in [72]. Various types of networks are discussed, such as consumer premises area networks (CPAN), home area networks (HAN), building area networks (BAN), industrial area networks (IAN), neighborhood area networks (NAN), and wide area networks (WAN). Various metrics are also listed (network latency, reliability, security, and time synchronization). In these networks, a consensus algorithm-based communication system was employed.

LoRa technology can be used in large outdoor/indoor areas such as offices, residential buildings, car parks, warehouses, etc., to understand their behavior and performance [73] in both the LoS (line-of-sight) and NLoS (non-line-of-sight) cases. The concepts of the path loss model, shadowing effect, and K-factor for the Rician model are presented. Further, in a multi-micro-grid environment, a deep neural network-based response learning mechanism for distribution system operators was introduced in [74]. These microgrids are linked to the main grid and acquire the power to match their local needs. Additionally, the pricing scheme was accomplished by reinforcement learning using the Monte Carlo method.

## 3. Insights into Communication Standards and Technologies

**Figure 4:**
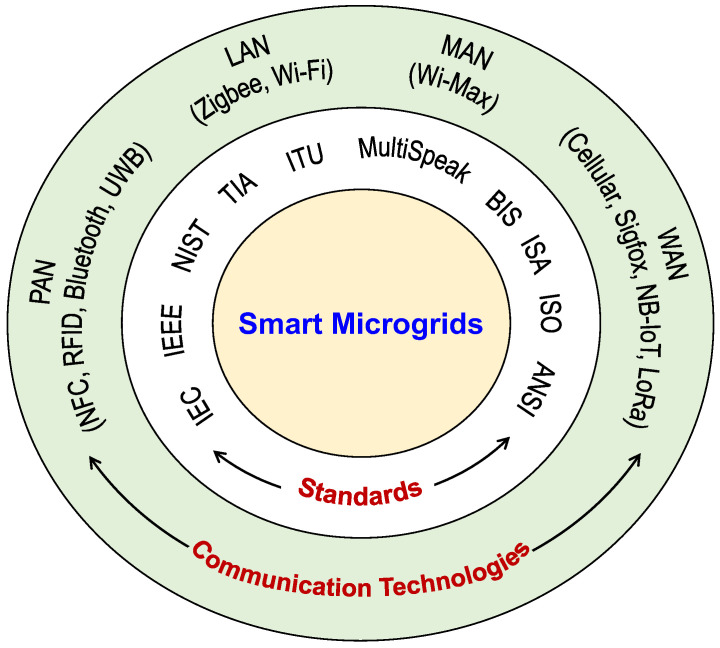
Key standards and technologies for smart microgrid communication.

As smart microgrids are one of the unique recent developments in the energy sector, their establishment requires a standard process to be followed. Thus, it is very important to understand various standards that contribute to the development of smart microgrids. Various renowned forums or agencies define different standards to be applied globally for the development of smart grids/microgrids. Further, to implement the architecture for the smart microgrid, one must have a detailed knowledge of the various technologies available. In the present-day scenarios, wireless communication technologies are outperforming wired-based solutions by offering advantages, such as mobility, convenience, easy installation, and low cost. All these key standards and technologies are noted in Figure 4, which are explained in the following subsections.

### 3.1. Standards and Guidelines

**Figure 5:**
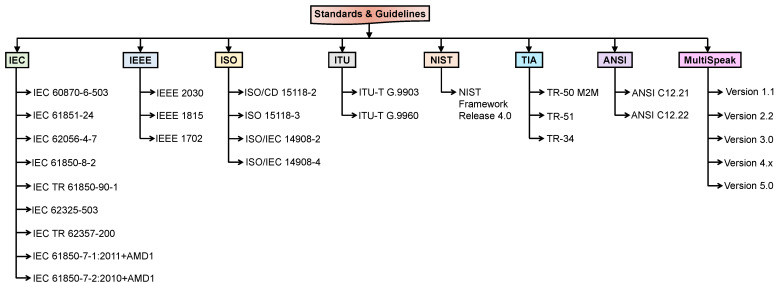
Detailed list of standards and guidelines for smart microgrid communication.

Standards provide procedures/detailed descriptions that can be followed universally. Governments and industries all around the world have recognized the importance of standards in the energy sector. Standards help to ensure quality and safety, and facilitate communication. In the absence of standards, it is difficult for manufacturers to design or implement a system which can be utilized globally. The smart grid does not just require a single standard; rather, its implementation will the use of several standards. Various standards and guidelines related to smart grid/microgrid communication are given in Figure 5 [75,76,77,78,79,80,81,82]. Some of the major contributors in this direction are IEC (International Electrotechnical Commission), IEEE (Institute of Electrical and Electronics Engineers), ISO (International Organization for Standardization), ITU (International Telecommunication Union), TIA (Telecommunications Industry Association), ANSI (American National Standards Institute), and MultiSpeak.

Usually, the standard documents are lengthy. It is therefore vital to organize these standards in a way that readers can quickly understand and select a standard based on their needs without going through the entire standard document. In this view, Table 6 helps the reader to understand the outline of these standards easily.

### 3.2. Communication Technologies

**Figure 6:**
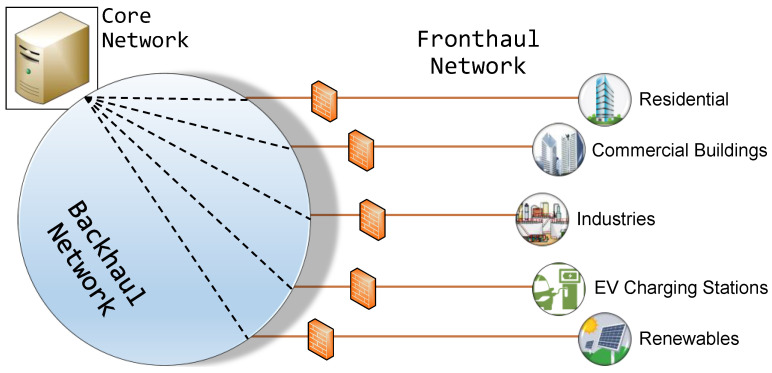
Typical connectivity scenario in the smart grid network.

To manage complex power systems effectively, information flow across various elements of the network is crucial. The typical connectivity scenario between various players involved in the smart grid is shown in Figure 6. Here, the entire network is built based on a fronthaul network, a backhaul network, and the core network. The fronthaul network connects end-users to the backhaul network. The backhaul network refers to the transmission of a remote signal from the fronthaul network to the central station (in the core network). This network consists of a high-capacity channel, which is capable of transmitting the data at higher data rates. The core network is a global network which interconnects the networks of different locations/systems, providing a path for the information exchange among them.

**Figure 7:**
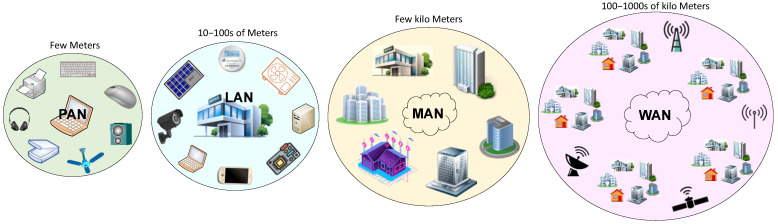
Elucidation of PAN, LAN, MAN, and WAN.

In the past, there was no communication between the generating station and the end-user. With the advancement of technologies in communication, now, bidirectional communication is possible, and thereby energy can be effectively managed between the generating station and the end-user. When compared to the traditional grid, the smart grid consists of several sensors and actuators. Sensors are used to collect data from various pieces of equipment with the help of controllers. The data can be sent to the server/cloud with the help of communication technologies. The collected data are used to analyze the performance of the equipment. Additionally, the actuators are used to control the grid components effectively. Generally, the system networks can be established in various fashions, such as personal area network (PAN), local area network (LAN), metropolitan area network (MAN), and wide area network (WAN), based on their distance of coverage, as shown in Figure 7. The PAN is a region where devices are connected within a person’s workspace (shorter distance). The major technologies used in PAN are Bluetooth, Radio-frequency identification (RFID), and Near-field communication (NFC). Bluetooth was developed by Bluetooth Special Interest Group and uses industrial, scientific, and medical bands (2.4 GHz). The latest Bluetooth Low-Energy (BLE) technology consumes little power by being in range. This technology is typically used to share files, images, videos, audio files, etc. The RFID technology is used to exchange information between the devices which are in close proximity. This works with the help of a tag and reader, where each tag is equipped with a unique number. This tag can be attached to the object (of desired application), which will be identified by the reader. Some of the applications are FASTag (toll collection system in India), tracking goods in warehouses, etc. NFC is a subset of RFID, but its range is even more limited. It is widely used in contactless credit card swiping and payments via mobile applications.

LAN is a group of interconnected devices that share information in a limited area such as an office, building, school, university campus, etc. The major technologies used in LAN are Zigbee and Wireless Fidelity (Wi-Fi). Zigbee is a low-cost, low-power wireless network that was designed as an open worldwide standard that works at low data rates. It works on the IEEE 802.15.4 standard and operates at 2.4 GHz. It is widely used in applications such as wireless sensor networks, home automation, etc. On the other hand, Wi-Fi provides higher data rates and coverage when compared to Zigbee but consumes more power. It works on the IEEE 802.11 standard and operates at 2.4/5 GHz. One of the popular applications of Wi-Fi is for accessing the internet. Users can establish a connection with access points, and these access points are connected to the Internet service provider (ISP) in the backend with the help of routers. The MAN is designed to connect the users that are spread across the metropolitan area; here, the coverage is higher. Worldwide Interoperability for Microwave Access (WiMAX) technology is used in MANs. This is a wireless broadband technology that works on the IEEE 802.16 standard. It is used in the applications of smart cities, i.e., to connect various offices, buildings, etc., that are located at multiple locations wirelessly.

The WAN is a communication network that extends the coverage over a large geographic area. The fifth generation of mobile networks, or 5G, is the latest version of the cellular technology that can perform at higher speeds than past generations, 1G, 2G, 3G, and 4G. The first generation, i.e., 1G, was in introduced in 1980s, where basic analog voice services are provided. In 2G, digital technology was used for voice calls (1990s). In 3G, mobile broadband was introduced (2000s), with the aim of providing good internet access, and in 4G, IP-based protocols were used (2010s). Now, 5G has been introduced, the main advantages of which are high speed, high bandwidth, and low latency [83]. This network splits the entire area into small regions known as cells. The cell is covered with the antenna with which all the 5G devices can be connected. The quality of Internet services in busy places can be improved by 5G since it is quicker than current networks and has a greater bandwidth. Further, 5G relies on network slicing, which means that multiple independent networks can use the same physical infrastructure. As this it is in the early stages of development, researchers and companies are working on various prototypes and real-time testing [84]. Additionally, in a few countries, it is already in use. 

Mobile-edge computing (MEC) offers execution resources such as storage, computations, etc., close to the users (in a network), that can be utilized to deliver services, as well as store and process the content. Artificial intelligence techniques help to further improve the performance of MEC [85,86]. Fifth-generation and MEC technologies together have the potential to greatly enhance performance and allow the real-time processing of large volumes of data. MEC lowers latency by bringing the processing capabilities closer to the user, while 5G improves speeds. Fifth-generation technology with MEC has created a new opportunity for industries to expand their business. These cellular technologies provide higher data rates but consume more power. LPWAN is a type of WAN designed to cover larger areas with low data rates and operates at low power. The major technologies competing in the LPWAN space are Sigfox, NB-IoT, and LoRa. Sigfox is a global network operator that connects various low-power devices, and here the downlinks are very limited. NB-IoT is developed by the 3rd Generation Partnership Project (3GPP) standard and operates in licensed bands. It uses a Long-Term Evolution (LTE) standard by limiting the bandwidth, whereas LoRa enables the devices to connect with bidirectional communication and operates at the unlicensed band. An individual can build a LoRa network without depending on the network operator. This means that the LoRa concept is adopted for most of the applications in LPWANs.

**Figure 8:**
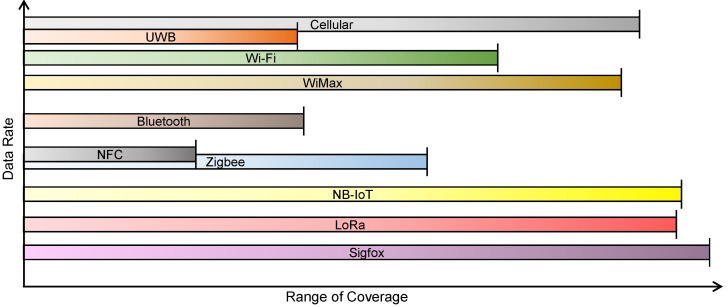
Comparison of various wireless communication technologies.

All these technologies can be arranged as shown in Figure 8 with respect to the range of their coverage and data rate support. Thus, based on the application requirements, suitable communication technology has to be selected. 

The spectrum plays an important role in wireless communications as it is expensive and should be utilized effectively. The International Telecommunication Union (ITU) is the major player in allocating the bands globally. Out of all the frequency bands, some bands are left open, which means the user need not take any approval to utilize these bands. These are called “industrial, scientific and medical” bands. These bands are given by ITU radio regulations, as given in Table 7. Additionally, users must follow the regulations set by the national/local government bodies (some bands may differ from the global perspective).

## 4. Issues and Challenges in the Implementation of ISMs

Electrical energy is a most demanded commodity across the globe which can cater for the needs of all sections of economical, industrial, and domestic sectors. So, it is expected to be easily accessible and readily available as per the requirements. However, many parts of the world still do not have the access to the grid. Further, the grid outages and uncertain renewable energy sources enhance the issue of energy availability. As a solution to these issues, ISMs are evolving worldwide. 

**Figure 9:**
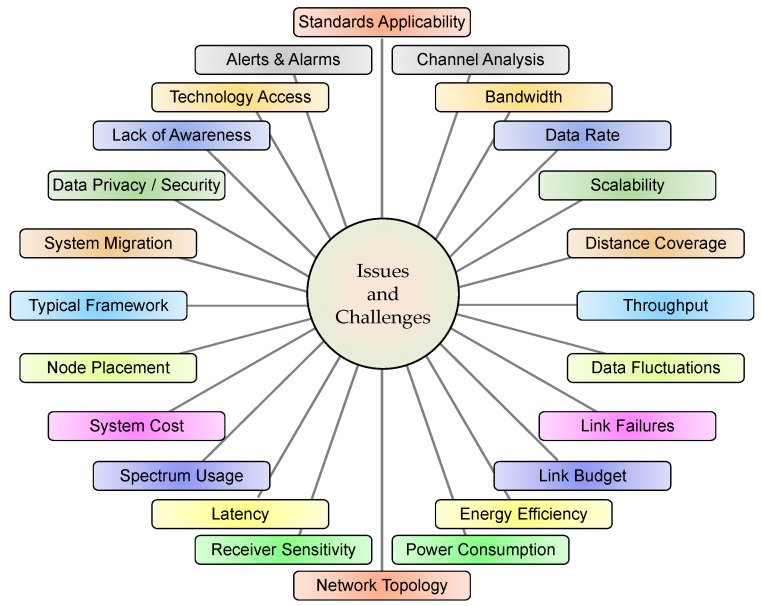
Key issues and challenges in ISM deployment.

These systems are expected to have functionalities/features such as effective generation-load balancing, economic load dispatch and unit commitment, robust communication infrastructure for parameter sensing and transmission, CMCU with data analytics and contingency estimations, decision and control operations for economic resource management, demand response, and advanced information technology solutions for effective data processing. However, many parameters have to be considered and issues/challenges need to be addressed for fruitful ISM deployment [8,87,88]. All these are depicted in Figure 9 and are summarized in Table 8.

## 5. Research Perspectives

**Figure 10:**
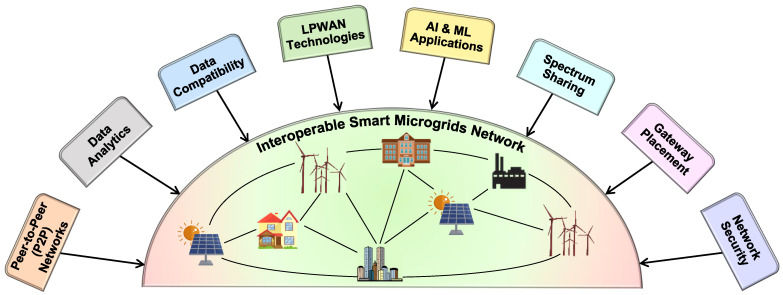
Future research directions for ISMs.

Though various works have been carried out on the implementation of ISMs, there is still much scope to increase the performance of these networks [9,10,11,89,90]. In this view, this section presents various possible research directions. All the possibilities are broken up into eight directions, as shown in Figure 10. These are outlined as general perspectives and emphasize LPWAN.

### 5.1. General Perspectives

As the ISMs are an emerging consideration for modern smart grids, it is required to adopt various advanced and highly efficient technologies and engineering concepts for their betterment. In this view, this section outlines the general perspective of using various technologies/concepts as follows:

▪ Peer-to-Peer (P2P) Networks: Peer-to-peer networks are still in the basic stages of development for use in microgrids, and further research is required. To establish the electrical exchange between peers in the community, robust communication is essential. In case of central monitoring station failures, peers can connect with each other without depending on a separate server. The study of microgrid dynamics is becoming more sophisticated as electrical systems become more complex and unpredictable, posing new challenges that must be addressed.
▪ Data Analytics: Data analytics is a powerful tool that helps to take the effective decisions in the operations. Having data alone cannot solve the purpose unless its analyzed. Users can benefit from data analytics due to the increased significance and the vast amounts of knowledge it generates. With the analysis of data, ISMs can find new opportunities. This results in wiser decisions, more effective operations, and more profits, which in turn helps in reducing the overall cost.
▪ Data Compatibility: Compatibility refers to the ability of systems to work together without requiring any modifications. An increasing number of renewable energy sources, loads and their integration to the microgrid generates varieties of data which requires advances in high-performance computing for computations. As a result, data compatibility is anticipated to be an important aspect in the deployment of ISMs. Additionally, another research aspect is defining the globally compatible communication protocols that can handle various data formats.
▪ LPWAN Technologies: These technologies are modern and advanced wireless communication technologies which provide wide-area coverage with massive volumes and offer low data rates. These technologies are emerging and offer solutions to the problems in IoT applications. Typically, these networks consume low power and in most cases they are battery-powered. Some of the popular technologies used in LPWAN are Sigfox, NB-IoT, and LoRa. As these are new technologies, the usage and applicability of these technologies for the establishment of ISMs have to be explored in greater detail.
▪ AI and ML Applications: Artificial intelligence (AI) and machine learning (ML) concepts can assist ISMs in building a robust communication network by learning from their previous actions and making better decisions. The algorithms influence the parameters such as channel bandwidth, antenna sensitivity, and spectrum monitoring. The backhaul network performance can also be further improved with the help of novel algorithms. Further, AI and ML concepts can be used for renewable energy resource forecasting, loading uncertainty estimation, cyber-attack prediction, the predictive maintenance of the ISMs, predicting channel behavior, predicting packet losses and network collisions, etc. Some of the latest and key applications of AI and ML in power grids are mentioned in Table 9.
▪ Spectrum Sharing: To make the overall communication system effective, the spectrum is one of the important aspects that needs to be managed carefully. If the system is not able to utilize the available spectrum (or part of the spectrum is not utilized), it is a huge loss. In such scenarios, spectrum sharing helps to enable the efficient utilization of the entire frequency spectrum so its full potential can be achieved. With the help of spectrum sharing, the operational cost for the telecom operators will go down, and thereby users can experience better quality and high speeds at a reasonable cost. The advancement in deep reinforcement learning algorithms helps to ensure intelligent spectrum access for users [101,102].
▪ Gateway placement: A judicious deployment of gateways helps to obtain the maximum data at CMCU from several motes in ISMs. The reception of the signals is different in cluttered environments compared to normal scenarios. LOS and NLOS paths play an important role in the reception of signals. Various new algorithms/metrics/methods have to be adopted for determining the best gateway placement in different environments, and thereby the data from the motes can be received at CMCU without any interruptions.
▪ Network Security: The data transfers among the motes and CMCU must take place in a secure environment. Network security safeguards the network infrastructure by preventing a wide range of potential threats from entering or spreading within a network. There is a possibility for the attacker to enter into network/network device (ex: switch) and create a malfunction in the network; thereby, it may lead to an entire network crash. Though there is a lot of research work carried out in the direction of security, attackers are coming with up new methods of attack. So, there is a necessity for researchers to propose new methods/approaches to prevent these new attacks. Furthermore, when developing information-exchange algorithms and protocols, users’ privacy should be prioritised.

### 5.2. Emphasis on LPWAN

Of the various points mentioned in Section 5.1, LPWAN is an advanced communication network that can meet various communication requirements. In recent years, there has been a huge shift in the market for LPWAN applications. This was proposed to meet the diverse needs of low-power and long-range IoT applications. These applications include smart metering (gas, water, etc.), smart cities, geolocation, asset tracking, etc. Battery-operated devices are used to gather data from different locations. There were several wireless technologies discussed in Section 2 of this paper, in which Zigbee was one of the most widely used conventional technologies for low-data-rate and long-distance communications. However, it cannot achieve coverage in the order of kilometers. To address this, in recent years, many new technologies have been invented for LPWAN.

In view of all these points, smart grid researchers need to understand how these new technologies can help in achieving their goals. With this objective, this subsection describes all such important technologies of LPWAN, viz., Sigfox, NB-IoT and LoRa. These advanced technologies are to be used to develop effective communication in ISMs which operates on different bandwidths and distances. The description of these suggested technologies and their features are given as follows.

**Figure 11:**
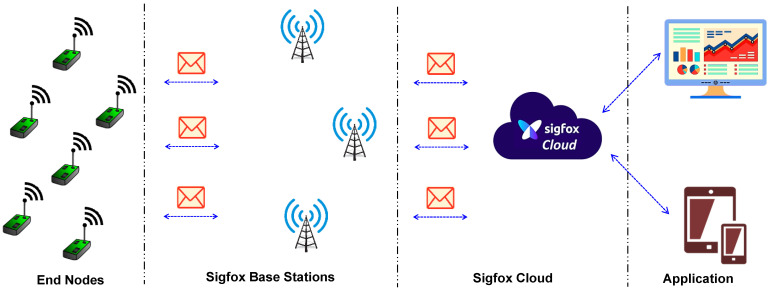
Sigfox network architecture.

Sigfox is a cellular-type network operator founded in 2010, with the objective of connecting devices in the physical world to the digital realm [103]. Currently, Sigfox is providing services in 75 countries. With the recent advancements in technology, people will depend on 5G, which provides very high speeds and bandwidth. The Sigfox 0G network will serve as a safety net and ensure that the devices are always in contact. This was created as a global IoT network, based on low power, long range, and small amounts of data, that provides end-to-end communication. With the help of the lightweight protocols, these devices consume very little power, and thereby devices can operate on battery power for long periods of time. As shown in Figure 11, the devices (sensor nodes) send the data from the remote locations to the Sigfox base stations, and the base stations will forward the data received to the Sigfox cloud. From the cloud, the data can be accessed by the user via the applications. The volume of the data that can be sent to the network (uplink) is 12 bytes (max), whereas the downlink data are restricted to 8 bytes (max).

**Figure 12:**
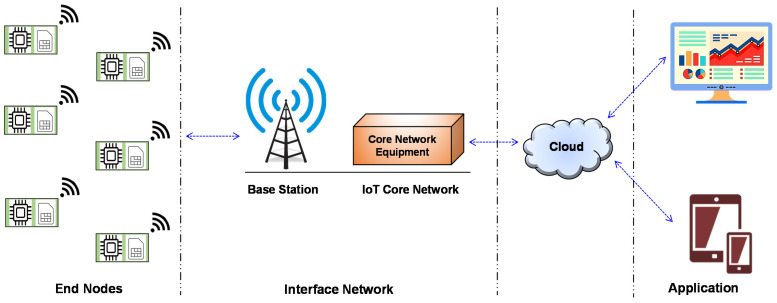
NB-IoT network architecture.

NB-IoT is a cellular device service technology that is established by the 3GPP for LPWAN. It works in a licensed band and can be developed on top of the existing cellular network infrastructure. It takes the advantage of mobile networks’ security and privacy characteristics. Today’s cellular networks provide high data rates but consume lots of power. On the other hand, NB-IoT allows the transmission of a smaller amount of data at a low data rate. When compared to general cellular networks, NB-IoT offers lower costs and a longer battery life. The three deployment modes for NB-IoT are in-band, guard-band, and standalone [104]. In in-band operation, it uses the frequency of the LTE channel, in guard-band operation it uses the unused guard bands, and in standalone mode, it occupies the GSM channels. The architecture of the NB-IoT network is shown in Figure 12. The function of the NB-IoT network is to send the end node data to the application server. The application server further processes the data and, based on the requirement commands, these can be sent to the end nodes from the application.

**Figure 13:**
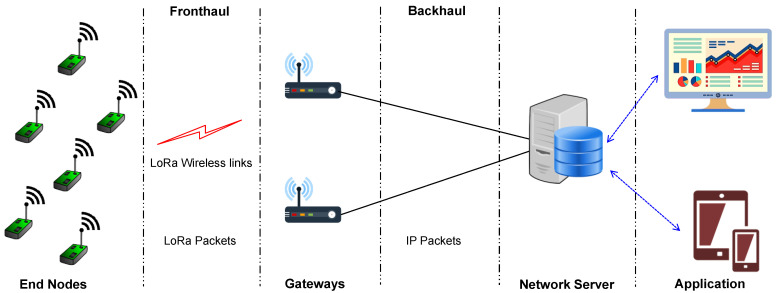
LoRa network architecture.

LoRa is a proprietary radio modulation technology based on the Chirp Spread Spectrum (CSS), owned by Semtech [105]. It is a PHY layer of the stack. LoRa provides a high link budget, allowing the receiver to receive signal levels below the noise floor. Higher Spreading Factor (SF) values spread the signal over more time, putting more energy and enabling fruitful reception over longer distances. The LoRa Alliance developed LoRaWAN, an open protocol standard built on top of LoRa [106]. A star-of-stars topology is used to implement the LoRaWAN network architecture. The end nodes send the data to the nearby gateways (LoRa packets), and the gateways further send the data to the network server via regular IP connections (IP packets), as shown in Figure 13. Further, the data from the network server are sent to the application server, where the user can access the data. All these three LPWAN technologies are compared considering various key aspects, as shown in Table 10 [13,107,108,109]. The range mentioned in Table 10 is ideal, and in real-time scenarios the range will be affected by several factors.

## 6. Proposed ISM Architecture Using LoRa Technology

From Section 5.2, among all the discussed LPWAN technologies, LoRa possesses certain unique characteristics for building standalone networks, where an individual can build their network without depending on the third-party service provider. Due to this unique advantage, LoRa finds usage in many present-day advanced communication networks-based applications. With this motivation, the following subsections provide more insights into the usage of LoRa technology with a proposed architecture for ISM development.

### 6.1. Dissecting LoRa

**Figure 14:**
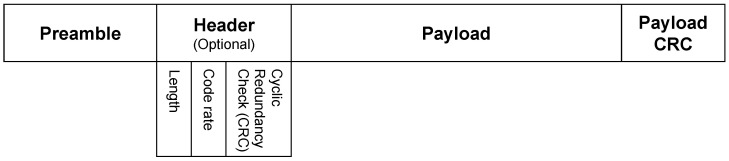
LoRa packet format.

LoRa technology is invented by Cycleo; Semtech later acquired Cycleo. Now Semtech has the intellectual property rights for LoRa. LoRa is available for two layers, namely the PHY layer (LoRa Radio) and MAC Layer (LoRaWAN). LoRa modulation is a unique spread spectrum technique, which uses wideband linear frequency-modulated pulses whose frequency changes over time. This makes LoRa immune to multipath fading and the Doppler effect. To represent data, LoRa uses up-chirps (increase frequency) and down-chirps (decrease frequency). It follows a packet format that is shown in Figure 14. To ensure security, an “advanced encryption standard” is used. The spreading factor, bandwidth, and coding rate are the key physical layer parameters that influence LoRa’s coverage and power consumption, which are described in the following [110,111]. 

**Figure 15:**
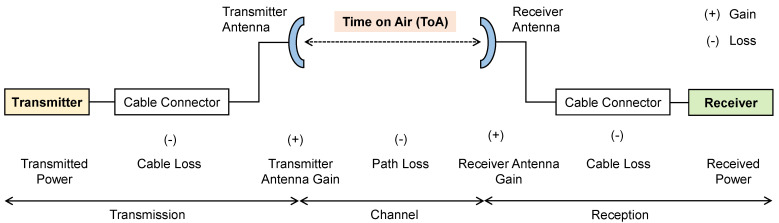
ToA representation in a transmitter–receiver communication system.

The number of bits used to represent each symbol is determined by the spreading factor. It is in the range of 7 to 12 depending on the application. According to the regional specifications document (for India), the bandwidth offered is 125 kHz and the data rate is 0.3 to 50 kbps. To achieve different data rates, “adaptive data rates” can be used as per the considered application. The forward error correction helps to recover the data during the interferences. This is achieved by adding the redundant bits. The coding rate is the portion of transmitted bits that carries the actual data. The code rates that are used by LoRa are 4/5, 4/6, 4/7, or 4/8. A greater code rate provides more protection; thereby, recovery becomes easy, but it also increases the Time on Air (ToA). When the transmitter antenna sends a signal, it takes a certain amount of time in air to reach the receiver antenna, which is called ToA and can be understood in Figure 15.

**Figure 16:**
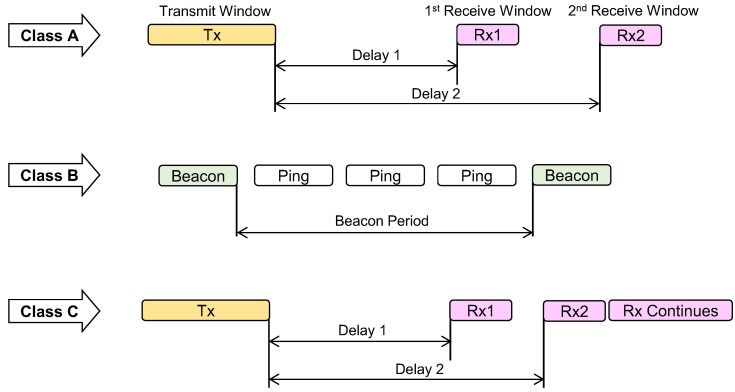
Tx/Rx windows for Class A, Class B, and Class C devices.

Any MAC layer can be used with the LoRa physical layer; however, LoRaWAN is the currently proposed MAC that uses a star-of-stars topology. It describes how end devices should communicate with the gateway. The gateways are then connected to network and application servers. Communication between the end node and the gateway may not be required at regular intervals in most real-time scenarios, so LoRaWAN considers three different categories of end devices, as shown in Figure 16, namely Class A, Class B, and Class C. The transmission can be started by the end node at any time in Class A devices. It opens two receive slots (Rx1, Rx2) after each transmission slot (Tx). Downlink transmissions can be accepted by Class B devices during scheduled receive slots. In Class C, the receiver slots will always be open, except during the uplink period. Devices will be listening to the gateway all the time [112,113,114]. Some of the latest and key works that are developed using LoRa are mentioned in Table 11.

### 6.2. Proposed LoRa Based Architecture

**Figure 17:**
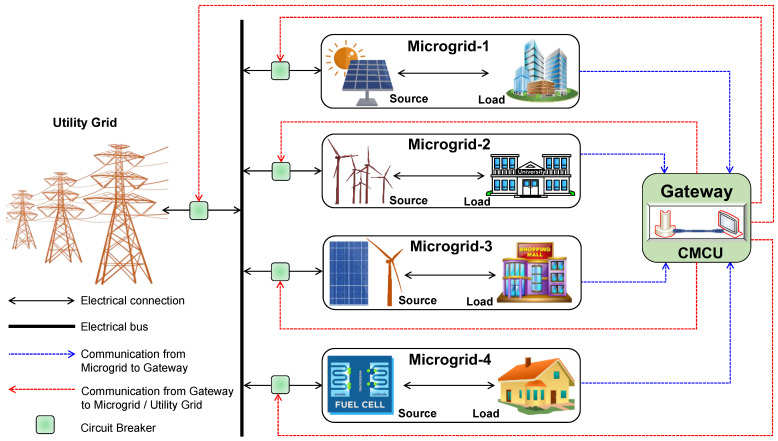
High-level view of ISM architecture.

To achieve the fruitful establishment of ISMs, an integrated architecture is proposed, as shown in Figure 17. To represent the concept of the proposed ISMs, four microgrids are interconnected with each other using a common electrical bus and communication network (through a gateway). Each building has its own local generation and load. This interconnected operation enables energy sharing among the microgrids. Further, the CMCU captures the energy generation and consumption data of each microgrid based on a predefined time interval and maintains this database for further actions. These actions involve energy management operations to optimally share the energy between the loads of various microgrids, performing various data analytics to study various data quality issues as well as a future estimate for contingency readiness, etc.

**Figure 18:**
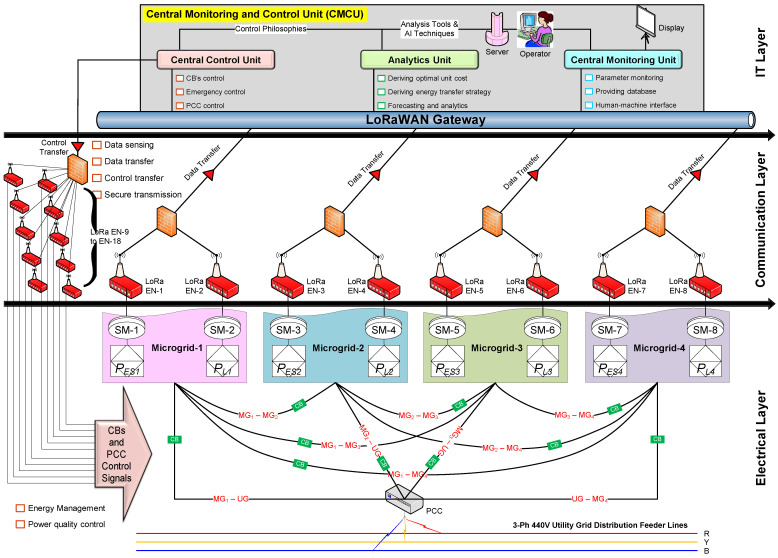
Three-layer representation of ISM architecture.

From an operation point of view, the entire ISM architecture shown in Figure 17 can be represented as a three-layer structure, as shown in Figure 18. It involves the electrical layer, communication layer, and IT layer.

The typical duties of the electrical layer are: facilitating energy management operations and power quality control. This layer has all the electrical equipment, viz., generating the sources and loads of each microgrid, electrical connectivity among microgrids and utility grid through circuit breakers (CBs). These CBs are operated based on the control commands sent by the CMCU. Further, it has metering devices (smart meters—SMs) to read the generation (PES1…n) and load (PL1…n). All the microgrids are connected to the utility grid at a node called the “point of common coupling (PCC)”.

The typical duties of the communication layer are facilitating continuous data sensing and data transfer from LoRa end nodes (ENs) to CMCU, control signals from CMCU to CBs and PCC, and providing secure transmissions between the electrical layer and IT layer. The ENs are connected to all the SMs (of sources and loads of each microgrid) and CBs. From these end nodes, the collected data will be transferred to the CMCU through the LoRaWAN gateway. Data security algorithms can be implemented to ensure security for the data transmission in both directions.

The IT layer has CMCU and LoRaWAN gateway. The CMCU operations are divided into three subunits, viz., central monitoring unit (CMU), analytics unit (AU), and central control unit (CCU). CMU has to monitor data continuously and provide a human–machine interface to supply user inputs. It also has to provide a database for storing the analysis results in future. AU has to perform all the computations and suggest optimal unit commitment for energy transfer between microgrids. It also has to perform forecasting for future contingency analysis and readiness and fault studies.

Additionally, the AU has to perform various data analytics operations using advanced machine learning algorithms to ensure a better visibility of the captured data, which helps to effectively operate the entire system. CCU has to provide the control signals to operate CBs or PCC for power exchange based on the optimal strategy derived by AU. It also has to provide alerts whenever any emergency or abnormality is detected.

**Figure 19:**
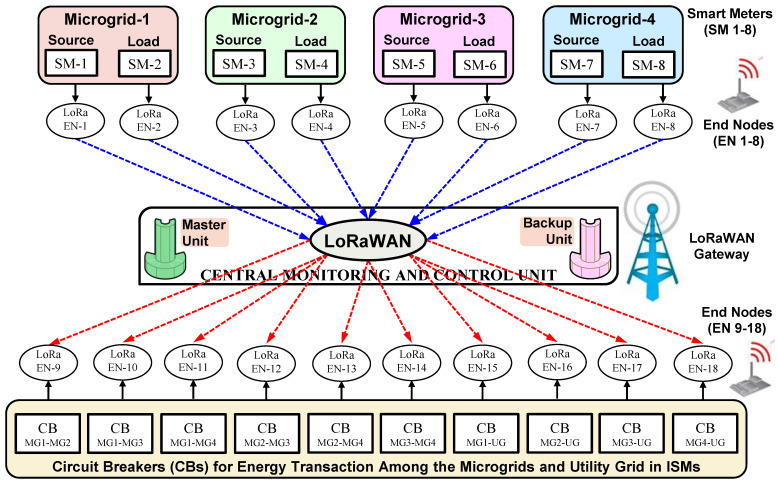
Low-level view of LoRa-based communication network for ISMs.

The low-level view of a LoRa-based communication network for the interoperability (interconnection) of various microgrids in a locality is shown in Figure 19. As shown in this figure, each of these buildings has a LoRa end node, which acts as a communication facility module for the corresponding building. As discussed above, the SMs capture the generation and load at the associated microgrid and communicate this information (via their respective LoRa end node) to the CMCU (through LoRa Gateway). According to these captured values, the CMCU computes the requirement of power export or power import for each of these microgrids based on the predefined energy management strategy. Based on these computations, the CMCU operates the corresponding CBs (via their respective LoRa end nodes) that are connected between the microgrids, to enable their interconnectivity. This interconnectivity through CBs facilitates the power import/export between any two microgrids, as dictated by the CMCU through respective control commands.

As shown in Figure 19, the CMCU has to be equipped with a backup computation (or storage) unit along with the master unit. This backup computation unit is a redundant unit, which ensures the protection of data under any failures of the master unit.

## 7. Summary and Outlook

The increasing importance of utility-grid independent systems in the present-day global energy sector encourages the study of various technologies on which these systems are relying. Keeping this in view, a systemic and technical assessment of various communication technologies that can be used for the development of ISMs is detailed in this paper. This paper started with a review of various state-of-the-art developments, where various worldwide footprints and Indian initiatives are discussed along with the work conducted at the macrogrid and microgrid levels. Then, it progressed to a discussion on various standards and communication technologies that are useful in deploying the ISMs. A discussion on numerous issues and challenges in the implementation of ISMs is provided.

Further, it is expected that this field of research will continue to expand, and with this theme, this paper suggests a number of possible research directions that could serve as a guide for researchers. A new communication strategy is needed to facilitate the transition from traditional centralized systems to decentralized systems. With the goal of improving the performance of communication networks for their use in microgrids, various advanced wireless technologies in LPWANs, such as Sigfox, NB-IoT and LoRaWAN, are detailed. At last, all these technologies are compared with respect to various features, and finally, this paper suggested a LoRa-based architecture for the ISMs with all necessary functionalities to establish effective ISMs.

### Limitations

We present here a few challenges that should be taken into consideration while implementing the proposed architecture:

▪ LoRa technology is limited in terms of its data rate. So, it may affect communication during emergencies, where fast data communication is normally desired.
▪ Grid connection and control in the microgrid network need to be developed in a robust manner as they may affect the entire power network.

## Figures and Tables

**Table 1:** Summary of review works presented in the literature.

Reference	Discussed Topics	Grid Level	Year
[7]	Current and Future Communication Solutions for Smart Grids.	Macrogrid	2022
[8]	Communication Technologies for Smart Grid.	Macrogrid	2021
[9]	Technical features of communication technologies, various standards and applications.	Macrogrid	2019
[10]	Latest advances in communication and information technologies.	Macrogrid	2018
[11]	Communication architectures challenges and future trends.	Microgrid	2018
[12]	Various elements of power system resilience.	Microgrid	2017
[13]	Various technologies and standards in the LPWAN (Low Power Wide Area Network).	Macrogrid	2017
[14]	Telecommunication technologies (wired and wireless).	Macrogrid	2016
[15]	Electrical layer and communication layers of microgrid.	Microgrid	2015
[16]	IEEE-1547, ISA-95, NIST, and IEC-61850.	Microgrid	2015
[17]	IEEE 1547.3.	Macrogrid	2015
[18]	Power systems, power electronics fields for microgrids, and potential avenues for further research.	Microgrid	2011

**Table 2:** Summary of worldwide initiatives.

S. No	Name/Title	Country	Description	Characteristics	Payment Policy/ Funding	Ref.
1	Carpathian Modernized Energy Network (CARMEN)	Hungary and Romania	Digitalization of the system with the deployment of communication and IT facilities	Infrastructure of 40 HV/MV modern transformer stations in 6 counties in the north-east region of Romania, in addition to a few more updates	Project promoters are the DELGAZ GRID, Romanian DSO, with support of the Romanian TSO and others	[19]
2	Gabreta SG	Czech Republic and Germany	This project introduces cross-border interconnections with the goal of the modernization and digitalization of energy infrastructure	Construction of Medium-Voltage (MV) and Low-Voltage (LV) lines with smart elements	German DSO Bayernwerk Netz GmbH (BAG) and the Czech DSO EG.D (EGD)	[20]
3	Accelerating Renewable Connections (ARC)	United Kingdom	Connecting several renewable energy sources quickly to the distribution network	Smart network management; integration of large-scale renewables	Scottish Power Energy Networks	[21]
4	ATENEA Microgrid	Spain	Facilitating the integration of renewable sources at the distribution level	The energy management software CeMOS^®^ is established to control and operate the entire system	CENE-National Renewable Energy Centre	[22]
5	BMW (Bear Mountain Wind) farm	Canada	34 ENERCON wind turbine generators were used	102 MW capacity	Owned by Bear Mountain Wind LP	[23]
6	BCIT (British Columbia Institute of Technology)	Canada	Solar and wind turbines were used	8KW of electricity	ICE (Innovative Clean Energy) NSERC CRD Grant	[24]
7	Afghanistan’s Bamiyan Renewable Energy Program (BREP)	Afghanistan	PV is used	1 MW capacity	Pre-paid pay-as-you-go model	[25]
8	Hydro powered minigrids	Nepal	2000 hydro-powered minigrids were installed with the help of AEPC (Alternative Energy Promotion Center)	30 MW capacity	Tariffs are not uniform	[26]
9	Hydro powered electrification Project	Tanzania	This project supported more than 2200 rural customers with a total of 21.5 GWh of AC power	4 MW capacity	Different tariffs available (lifeline, wholesale)	[27]
10	Rural electrification in Kigbe, Nigeria	Nigeria	Schneider Electric and Havenhill Synergy collaborated to build a minigrid	A local (onsite) minigrid was established	Fund from the United States African Development Foundation and Power Africa Initiative	[28]
11	Las Positas college	USA	This community college is located in Livermore, California	55% of the campus energy requirement is managed by the solar arrays	USD 15 million funds supported by California Energy Commission	[29]
12	ARC power minigrids	Rwanda	The 1st phase of an ambitious scheme to erect a large set of minigrids in Rwanda	PV (first phase: 0.12 MW; whole project: 3.5 MW)	Energy from minigrids is being offered on pre-pay, pay-as-you-go basis to off-grid communities	[30]
13	Buffalo Energy Ltd.	Zambia	Buffalo Energy Ltd. was established in 2016 to develop small-scale projects to provide renewable, low-cost power.	Solar PV, biomass, wind Estimate of capacity: 30 MW	REPP funding support	[31]
14	POWERGEN AND CBEA	Tanzania	As the operator, PowerGen will be incentivized to ensure the efficient operation of the sites.	PV installed capacity: 1.2 MW	Pay-As-You-Go basis	[32]
15	AEDB	Pakistan	Hydro, solar, and wind power are used	22 solar PV power projects with a cumulative capacity of nearly 890.80 MW	Government of Pakistan	[33]
16	Yokohama Smart City Project (YSCP)	Japan	Solar, wind, and hydro power and biomass are used	A goal was set on 20% energy reduction by 4000 homes	The consortium of seven Japanese companies	[34]

**Table 3:** Summary of Indian initiatives.

S. No	Name/Title	Description	Characteristics	Payment Policy/Funding	Ref.
1	Island minigrids in West Bengal	23 minigrids were developed by WBREDA (West Bengal Renewable Energy Development Agency)	Primarily solar photovoltaic is used along with other renewable technologies. Additionally, the range is between 25 kWp and 100 kWp	Flat fee for a fixed amount of power	[35]
2	Biomass gasification minigrid (primarily in Bihar)	Decentralized Energy Systems of India (DESI) installs biomass gasification minigrid systems	Capacity varies from 30 kW to 150 kW	Payment policy depends on the type of customer: commercial customers or residential customers	[36]
3	Baikampady mangalore microgrid (SELCO) in Karnataka)	75 houses were provided with power	1.2 kV PV panels are used	Charged based on the type of household	[37]
4	Kalkeri Sangeet Vidyalaya	This is a residential music school. Grant was awarded to the school	14 kW PV is used to manage the needs	The system was paid for in advance with a percentage paid on commissioning of the system	[38]
5	Darewadi solar microgrid, Pune district	Darewadi grid is maintained by the village committee	A solar capacity of 9.36 kW is available	Payments depending on electric usage	[39]
6	Bhamane hybrid (solar+ micro-hydro) microgrid	Bhamane is located in Uttar Karnataka District	Solar capacity—3 kW; hydro capacity—5 kW	Monthly payments depending on electric usage	[40]
7	Surya raitha scheme	BESCOM (Government of Karnataka) has taken up a pilot project	The pilot is taken up in Kanakapura Taluk for energizing 310 IP sets	Tariff is decided by KERC (Karnataka Electricity Regulation Commission)	[41]

**Table 4:** State-of-the-art literature works on macrogrids.

S. No	Objectives/Technology	Merits/Methods/Metrics	Year	Ref.
1	IEEE 802.14.4g, IEEE 802.14.4e	MR-FSK, MR-OFDM, and MR-OQPSK; Header IE and Payload IE	2012	[42]
2	Smart grid communication	Challenges such as ensuring standard interoperability, unlicensed radio spectra, and cybersecurity are discussed.	2013	[43]
3	Interoperability	Steps: (1) IT layer; (2) electrical layer; (3) communications.	2015	[44]
4	D2D communication	Information loss rate and peak bandwidth are used as metrics. The rules were made around relay nodes and base stations in methodology execution.	2016	[45]
5	Secured communication	A lightweight authenticated communication scheme is proposed.	2016	[46]
6	Telecommunications	Wired and wireless technologies are discussed.	2016	[14]
7	Smart meter data collection	VANET is used to collect data from wireless automatic meter reading.	2016	[47]
8	Spectrum sharing	Priority-based communication strategy is proposed.	2017	[48]
9	AMI for PLN Bali	LoRaWAN is proposed to collect data from meters.	2017	[49]
10	Smart sensors for smart grids	Phasor measurement unit-based smart sensors is developed. However, high-accuracy timing and time synchronization are constraints.	2017	[50]
11	Smart choice for smart grids	NB-IoT is discussed. However, its real-time deployment will be costly.	2018	[51]
12	LoRa communication	Distance, obstacles, and noise are used as metrics for this study.	2019	[52]
13	Insider threats detection	Novel hybrid insider threats model is proposed.	2019	[53]
14	Future communication and information infrastructures	Various standards and applications were discussed.	2019	[9]

**Table 5:** State-of-the-art literature works conducted on the microgrid.

S. No	Objective(s)	Merits/Methods/Metrics	Year	Ref.
1	Case studies in various countries	Power systems and power electronics fields of microgrids are discussed.	2011	[18]
2	Energy sharing between neighboring households	A local grid with a single power generation source is discussed. Communication between the households is given importance.	2013	[54]
3	State of the art in microgrid communication	Internet Protocol suite, DNP3, Modbus, and IEC 61850 are discussed.	2014	[55]
4	Frequency synchronization of several isolated microgrids	LTI system approach with consensus algorithm is used.	2014	[56]
5	Synchronization in microgrids with communication latency	Distributed secondary control algorithm is used.	2015	[57]
6	Zigbee for microgrids	Communication delay is taken as a metric.	2015	[58]
7	Security scheme	Control loop delay is the metric used for co-simulation.	2015	[59]
8	State of the art in microgrids	The electrical layer and communication layer are reviewed.	2015	[15]
9	Review on IEEE-1547, ISA-95, NISA, IEC-61850	Suggestions were given mostly for IEEE-1547 and IEC-61850 architectures. It requires two copies of diagnostic packets.	2015	[16]
10	Distribution system automation	Technologies and levels of automation are discussed.	2015	[60]
11	Home Energy Management System (HEMS)	IoT-based HEMS with a PV system is proposed. A nomadic agent is used to achieve DHANs.	2015	[61]
12	Communication and data acquisition	SunnyBoy Webbox, RedLion data logger, power meters, etc., are used.	2015	[62]
13	Various views of distributed resource system architectures	The architectural view, requirements view, conceptual view, concurrency view, and network view are discussed.	2015	[17]
14	Distributed power-sharing	Game theory approach is used.	2017	[63]
15	Synchronization of power inverters	FM signal is used as a synchronization signal.	2017	[64]
16	Reconfigurable control and self-organizing communication	Unique operational characteristics of CRMs are discussed.	2017	[65]
17	Networked microgrids	Various elements of power system resilience are discussed.	2017	[12]
18	Ethnography of electrification	Mutual energy exchanges are discussed.	2017	[66]
19	Internet of microgrids	Interconnection within microgrid and between microgrids is discussed.	2018	[67]
20	Energy management automation	Sizing and structuring of communication messages for energy management automation are discussed.	2018	[68]
21	Communication architectures for microgrids	It focuses on centralized (SCADA), decentralized (MAS), and distributed dynamic (P2P-Overlays) structures.	2018	[11]
22	SHS and smart meters for the smart grid are discussed	Packet loss of different messages, ETE delay, etc., are discussed.	2018	[69]
23	P2P energy sharing	Self-consumption, self-sufficiency, and energy cost are considered as the assessment metrics.	2018	[70]
24	Impact of ICT degradation	Latency for different wireless technologies was discussed.	2019	[71]
25	Microgrid communication system	Various technologies, categories, and metrics are discussed.	2019	[72]
26	LoRa technology in multi-floor buildings	K-factor for the Rician model is used for the analysis.	2020	[73]
27	Intelligent multi-micro-grid energy management	DNN and a Monte Carlo method are used.	2020	[74]

**Table 6:** Various standards and their scope for smart microgrid communication.

Standard Number	Description	Details	Year
IEC (International Electrotechnical Commission)			
IEC 61850-7-2:2010+AMD1	Communication networks and systems for the power utility automation—Part 7-2: Basic information and communication structure—Abstract communication service interface (ACSI)	(i) Interface for specifying communications between client and remote server (ii) Event distribution between the application of a single device and the application of several remote devices	2020
IEC 61850-7-1:2011+AMD1	Communication networks and systems for power utility automation—Part 7-1: Basic communication structure–Principles and models	The goal of this standard is to help people understand the basic modelling concepts and methods for: (i) information models which are substation-specific; (ii) device functions for power utility automation; and (iii) communication systems to achieve interoperability	2020
IEC 61850-8-2	Communication networks and systems for power utility automation—Part 8-2: Specific communication service mapping (SCSM)—Mapping to extensible messaging presence protocol (XMPP)	The important aspects in mapping are: (i) End-to-end security, (ii) describing the XML payloads, and (iii) describing the features of XMPP	2018
IEC 62325-503	Framework for energy market communications—Part 503: Market data exchange guidelines for the IEC 62325-351 profile	Advanced message queuing protocol (AMQP) is adopted	2018
IEC 62056-4-7	Electricity metering data exchange—The DLMS/COSEM suite—Part 4-7: DLMS/COSEM transport layer for IP networks	Connectionless and connection-oriented transport layers are discussed	2015
IEC TR 62357-200	Power systems management and associated information exchange—Part 200: Guidelines for migration from Internet Protocol version 4 (IPv4) to Internet Protocol version 6 (IPv6)	This standard addresses the issues concerned to migration from IPv4 to IPv6	2015
IEC 60870-6-503	Telecontrol equipment and systems—Part 6-503: Telecontrol protocols compatible with ISO standards and ITU-T recommendations-TASE.2 Services and protocol	Exchange of real-time data, control operations, scheduling and accounting information, remote program control, and event notification were mentioned	2014
IEC 61851-24	Electric vehicle conductive charging system-Part 24: Digital communication between a d.c. EV charging station and an electric vehicle for control of d.c. charging	Discusses the digital communication part between the EV charging station and electrical vehicle	2014
IEC TR 61850-90-1	Communication networks and systems for power utility automation—Part 90-1: Use of IEC 61850 for the communication between substations	The key points discussed are (i) communication requirements, (ii) services and architecture, (iii) interoperable prerequisites, and (iv) enhancements to SCL (substation configuration description language)	2010
IEEE (Institute of Electrical and Electronics Engineers)			
IEEE 1815	IEEE Standard for electric power systems communications-distributed network protocol (DNP3)	This standard uses distributed network protocol (DNP3) which specifies structure, application choices and various functions	2012
IEEE 1702	IEEE standard for telephone modem communication protocol to complement the utility industry end device data tables	This standard provides a “plug and play” environment for the various metering devices that are currently deployed in the field.	2011
IEEE 2030	IEEE guide for smart grid interoperability of energy technology and information technology operation with the electric power system (EPS), end-use applications, and loads	IEEE 2030 gives the roadmap for attaining smart grid interoperability by a smart grid interoperability reference model (SGIRM). It has the info of the electric power system with emphasis on evaluation criteria, characteristics, etc.	2011
ISO (International Organization for Standardization)			
ISO/CD 15118-3	Road vehicles—Vehicle to grid communication interface—Part 3: Physical and data link layer requirements	This standard provides the details of PHY and link layer to establish a high-level communication network (wired) between an electric vehicle and a charging station	2015
ISO/CD 15118-2	Road vehicles—Vehicle-to-grid communication interface—Part 2: Network and application protocol requirements	This standard discusses the communication establishment between electric vehicles and electric vehicle supply equipment (EVSE)	2014
ISO/IEC 14908-4	Information technology—Control network protocol—Part 4: IP communication	This standard aims to provide interoperability between different control network protocol (CNP) devices that want to interact over IP networks	2012
ISO/IEC 14908-2	Information technology—Control network protocol—Part 2: Twisted pair communication	The CNP free-topology twisted-pair channel for networked control systems in local area control networks is defined in this standard	2012
ITU (International Telecommunication Union)			
ITU-T G.9960	Unified high-speed wireline-based home networking transceivers—System architecture and physical layer specification	This standard recommends PHY layer functionalities for transceivers in the home network designed for communication using coaxial cables, PLCs, optical fibers, etc. Additionally, this standard specifies reference models and architecture	2018
ITU-T G.9903	Narrowband orthogonal frequency division multiplexing power line communication transceivers for G3-PLC networks	This standard recommends PHY and link layer specifications for G3-PLC transceivers	2017
TIA (Telecommunications Industry Association)			
TR-51	Smart utility networks	Smart utility network standards are designed to deliver better solutions for bidirectional communication between devices and the service provider’s backhaul systems	2012
TR-50 M2M	Smart device communications	This standard defines interface canons for communication between machine-to-machine (M2M) systems and other smart devices	2010
TR-34	Satellite equipment and systems	This standard emphasis is on satellite communication systems covering both space and terrestrial. The focus of this standard is mainly on (i) the optimal use of spectrum and orbital resources, (ii) spectrum sharing and (iii) interoperability between satellite systems	2001
ANSI (American National Standards Institute)			
ANSI C12.22	The protocol specification for interfacing data communication networks	This standard works to improve the interoperability between various meters and communication units for the data transmission	2012
ANSI C12.21	The protocol specification for telephone modem communication	The norms for communicating between a C12.21 device and a C12.21 client via a modem connected to a telephone network are detailed in this standard	2006
MultiSpeak			
Version 1.1, Version 2.2, Version 3.0, Version 4.x and Version 5.0	Standard for fulfilling enterprise application for interoperability at full potential	It mainly employs (i) common data semantics, (ii) message structure (syntax) and (iii) which messages are needed to support various processes in the business	2000

**Table 7:** Various industrial, scientific and medical bands.

S. No	Frequency Range	Acceptability
1	6.765–6.795 MHz	Subjected to local body regulations
2	13.553–13.567 MHz	Globally
3	26.957–27.283 MHz	Globally
4	40.66–40.7 MHz	Globally
5	433.05–434.79 MHz	Subjected to local body regulations
6	902–928 MHz	With few exceptions
7	2.4–2.5 GHz	Globally
8	5.725–5.875 GHz	Globally
9	24–24.25 GHz	Globally
10	61–61.5 GHz	Subjected to local body regulations
11	122–123 GHz	Subjected to local body regulations
12	244–246 GHz	Subjected to local body regulations

**Table 8:** Various key issues and challenges for ISM implementation.

Issue/Challenge	Description
System Knowledge - Standards applicability - Lack of awareness - Technology access - Typical framework	- People should endeavor to understand available communication technologies and their usage. Additionally, as the new wireless technologies are emerging day by day, it is always recommended to adopt standards defined by the statutory bodies so that new devices can be easily integrated into the existing infrastructure. Some cost-effective and simple technologies have been developed worldwide, but may not be available in many developing and underdeveloped countries. - Further, regulatory bodies such as IEEE, ISA, NIST, and IEC have defined various architectures for ISMs. It is very important to understand these architectures before the deployment of ISMs and select a suitable architecture for the location or application; thereby, various components can be interfaced effectively.
System Characteristics - System migration - Scalability - System cost - Alerts and alarms - Power consumption - Energy efficiency - Receiver sensitivity - Node placement	- Every few years, new technologies will be evolving in the market. So, transferring the business process resources to a newer hardware/software platform is essential. To move the current application to the new technology to ensure better business value, system migration is required. Additionally, the systems shall be scalable to enhance the business as per the new requirements. - On the other side, interoperability of new–old communication systems in an industrial scenario must be considered, where the existing systems may use one type of communication protocol and the newly installed one works on a different protocol. So, the integration and interoperation of these two will be difficult. Protocol converters can be used as a solution, but still there may exist an issue with data misinterpretation in the process of protocol conversion. - However, the investment costs for the system deployment with currently available advanced technologies is high. So, this became a constraint for many countries to implement smart distribution power networks. - Apart from the automated alerts and alarms, the networks will have to be facilitated with some manual configurations. So, to cater for this requirement, the communication technology shall be easy and understandable to the operators to program the manual alerts when necessary. - The quantity of energy utilized per unit of time is referred to as power consumption. It is always desired to establish a communication mechanism with low power consumption for data transmission. Thus, while designing the network components for ISM implementation, power consumption is an important aspect to be considered. As the nodes in the network are powered by batteries, energy efficiency is critical. In particular, when the number of mobile devices increases in the network, energy management will become a major concern. To overcome this issue, solar-powered systems and optimum scheduling algorithms can be used. - Receiver sensitivity is the lowest signal level from which the receiver can sense the signal. The receiver with the highest receiver sensitivity will have the capability to receive the weak signals. If the received signal strength is lower than the receiver sensitivity, then the receiver will not receive the data. Some of the key factors that influence sensitivity are thermal noise, signal to noise ratio, and noise figure. - In wireless environments, the reception of the signal from the microgrid also depends on the receiver’s location. To receive the best signal, optimum node placement is an important aspect to be considered while implementing the ISMs.
Network Characteristics - Channel analysis - Network topology - Latency - Distance coverage - Link failures - Link budget - Spectrum usage	- Channel is a part of the medium which is used for the establishment of communication between the transmitter and the receiver. Before the connection establishment, it is very important to understand and analyze the channel characteristics, so that modifications can be made to the transmitting signal to ensure minimal losses during the transmission. Suitable channel modelling and selection will have to be conducted in consideration of the distance of coverage. - Network topology assists us in better comprehending networking principles. Small-scale network deployments that can adapt to varying levels of traffic have proved to save energy while maintaining great service quality. The network topology should also handle the expansion while responding to areas with varying traffic demands. So, the network topology has a significant impact on performance. - High fidelity for emergency operations and islanding while giving instructions to operate control systems appropriately in emergencies (e.g., occurrence of faults, severe disturbances, etc.), the communication medium should be very fast and robust to quickly perform islanding operations. The same has to be ensured when performing operations such as load balancing, demand-/source-side management, demand response, etc. Further, while tracking second-by-second data in the proposed scenarios, such as urban community ISMs, the data available will be huge, and have to be transmitted to central control rooms to take necessary decisions for power exchange. So, handling these big data with effective communication is required. - Sometimes, link failures can be observed during the communication; these may occur because of misconfiguration, system vulnerabilities, issues in the channel, etc. A backup mechanism must be taken into consideration during the link failures so that the data can be sent to the destination without any loss. - Link budgeting is an accounting of all the power gains and losses that a communication signal encounters. While designing the system, the link budgeting calculations should be carried out properly to receive the signal with a good signal-to-noise ratio. Some of the factors that influence the link budget are antenna losses, terrestrial interferences, etc. - The term “spectrum” refers to a range of electromagnetic radio frequencies that are utilized to transmit voice, data, and images. Spectrum is a highly precious resource in wireless communications. Though electromagnetic waves are invisible, their role is crucial. Therefore, spectrum management should be carried out effectively.
Data Capturing and Analysis - Bandwidth - Data rate - Throughput - Data fluctuations - Data privacy/security	- While designing an ISM network, the parameter calculations such as bandwidth, data rate and throughput play an important role. These metrics should be managed carefully to maximize the performance of the network. - The data would fluctuate greatly due to the unpredictable nature of renewable energy sources. So, sensors have to be well adapted to those changes, which has a great impact on communicating correct information. Additionally, the communication link failures create data loss, which can give wrong or improper data analytics. - Data privacy and security should be a part of ISMs’ architecture. The service provider/central coordinator will have the personal information of all the stakeholders of the ISM. It is essential for the service provider to protect the stakeholder’s data. A unauthorized person should never be given control over. Legal frameworks should be maintained.

**Table 9:** Key applications of AI and ML in power grids.

Objectives	Technique Used	Key Points	Year	Ref.
Power system state classification	Supervised learning using AdaBoost	Accuracy, mean square error, false-negative rate, false-positive rate, computational time	2011	[91]
Future smart grids	AI	Two-layer simulation framework was proposed	2014	[92]
Social network concept to smart grids	Support vector machine (SVM)	Humidity, rainfall, atmospheric pressure, sun time	2014	[93]
Energy management system (RLbEMS)	Batch Reinforcement Learning	Energy generation, consumer demand, energy prices, characteristics of storage systems were mentioned	2015	[94]
MAS (Multi-agent system) for power grid communication	AI	REQUEST, SUBSCRIBE, CONFIRM, INFORM, and CFP are used	2016	[95]
Convergence of machine learning and communications	Machine Learning	Communications, security, privacy	2017	[96]
Cyber deception assaults	FS-based SVM scheme	Accuracy and F1 score are used	2018	[97]
ML techniques for smart grid applications	SVM, Descriptive Discriminant Analysis, Decision Trees and Neural Networks	Precession, accuracy, linearity, training time, frequency of use, etc.	2018	[98]
Various security concerns	Big data and ML techniques	Various attacks such as spoofing, tampering, information disclosure, etc.	2019	[99]
Deep learning in smart grids	Deep learning	Feature extraction and handling a huge amount of data	2019	[100]

**Table 10:** Comparison of key parameters of Sigfox, NB-IoT, and LoRa technologies.

Feature	Sigfox	NB-IoT	LoRaWAN
Operating Frequency	868 to 869 MHz and 902 to 928 MHz (depending on region)	LTE Frequency Bands	IN865-867, US902-928, EU433 and EU863-870 (depends on region)
Licensed/Unlicensed band	Unlicensed band	Licensed Band	Unlicensed band
Type of modulation	BPSK	QPSK	CSS
Bandwidth	100 Hz	200 kHz	125 kHz, 200 kHz
Coverage	3–10 km (Urban) 30–50 km (Rural)	1–5 km (Urban) 10–15 km (Rural)	2–5 km (Urban) 15–20 km (Rural)
Standard developed by	Sigfox in collaboration with ETSI	3GPP	LoRa Alliance (Network)
Data rate	100 bps	200 kbps	50 kbps (adaptive)
Sensitivity to interference	High	Low	High
Network setup	By operator	By operator	Individual can setup their own networks

**Table 11:** Key works developed using LoRa technology.

Objectives/Technology	Key Points	Year	Reference
Relay network based on LoRa	Forwarding scheme based on broadcast scheduling is discussed	2020	[115]
Multi-hop relay and Automatic Repeat Request (ARQ)	Packet delivery rate is significantly improved with the help of multi-hop	2020	[116]
Coverage test for LoRa	Range is tested, on road (15 km) and on water (close to 30 km)	2015	[117]
Investigation on capacity limits of LoRa	Data Extraction Rate (DER)and Network Energy Consumption (NEC) are used	2016	[118]
Chirp Spread Spectrum (CSS)	BER, range and coexistence are discussed	2016	[119]
Review on LPWAN	All the technologies and standards in the LPWAN are discussed	2017	[13]
Bidirectional traffic in LoRaWAN	Duty cycle limitations, energy consumption, and reliability tradeoffs are discussed	2017	[120]
LoRa scalability	Performance of the network with respect to scalability is discussed	2017	[121]
Mathematical model of LoRaWAN	Packet error rate (PER) dependency with load is discussed	2017	[122]
Evaluating the sub-gigahertz wireless technologies	Improvement of LoRa message delivery ratio over Wi-Fi	2017	[123]
LoRaWAN channel modelling	A general model is developed which can be used to evaluate the performance of LoRaWAN	2017	[124]
LoRaWAN based AMI	LoRa WAN BTS location measures RSSI	2017	[49]
Improving LoRa performance with CSMA	CSMA (Carrier Sense Multiple Access), an enhancement to LoRaWAN that lowers the collision ratio is discussed	2018	[125]
LoRaWAN module is developed in NS-3	Class A type and LoRaWAN 1.0 are considered	2018	[126]
Channel modelling on the IIUM campus	Measurement tests of three different scenarios for both LOS and NLOS links conducted	2018	[127]
Performance evaluation of LoRa for different scenarios	Several scenarios for urban, suburban, and rural are considered	2018	[128]
Scalability concerns of CSS	Collisions and packet error rates are used to describe the effect	2019	[129]
E-Metering with LoRa	A smart water distribution system is implemented where each meter is connected to a mote	2019	[130]
Radio propagation models	The path loss model was considered	2019	[131]
S-Aloha on LoRaWAN	Network throughput is improved	2019	[132]
Bridge between IoT and smart grid	To check the feasibility of the APP, experiments were performed in 81 locations, from the PER values, APP is tested	2019	[52]
Modelling, characterization and measurement of LoRa	Energy consumption, characteristics, and coverage are discussed	2020	[73]

## Data Availability

Not applicable.
